# Trends in hospitalisation, myasthenic crisis and intensive care use in myasthenia gravis: a nationwide population-based study in Spain (2016–2022)

**DOI:** 10.1186/s42466-026-00485-5

**Published:** 2026-04-21

**Authors:** Tania Garrido-Hernández, Javier Del-Águila-Mejía, Marta Rodríguez-Camacho, María del Mar Martínez-Salmerón, Beatriz Vélez-Gómez

**Affiliations:** 1 Department of Neurology, Hospital Universitario Torrecárdenas, Hermandad de Donantes de Sangre S/N Street, Almería, 04009 Andalusia Spain; 2 National Center for Epidemiology, Carlos III Health Institute, Madrid, Spain; 3 Epidemiology and Public Health Biomedical Network Research Consortium (CIBERESP), Madrid, Spain; 4 Centro Investigación Biomédica en Red Enfermedades Neurodegenerativas (CIBERNED), Instituto de Salud Carlos III, Madrid, Spain

**Keywords:** Myasthenia gravis, Hospitalization trends, Myasthenic crisis, Hospital admissions, Ambulatory care, Population-based study

## Abstract

**Introduction:**

Myasthenia gravis (MG) is a chronic autoimmune disorder increasingly affecting older adults, with rising prevalence and comorbidity burden across Europe. Contemporary nationwide data describing MG-related hospital utilisation in European healthcare systems remain limited. We examined temporal trends and severity-related hospital outcomes in Spain between 2016 and 2022.

**Methods:**

We conducted a retrospective, population-based analysis using the Spanish Minimum Basic Data Set, including all adult hospital episodes with a primary diagnosis of MG (ICD-10-ES G70.00, G70.01). Hospital admissions, day-hospital visits and emergency department (ED) encounters were evaluated. Variables included demographics, intensive care unit (ICU) admission, myasthenic crisis, in-hospital mortality and comorbidities. Temporal trends were analysed using segmented negative binomial regression models incorporating a breakpoint in 2018.

**Results:**

A total of 20,251 MG-related episodes corresponding to 6,308 unique patients were identified. Following 2018, hospital admissions declined by 5.4% annually (95% CI − 8.4 to − 2.4), whereas day-hospital and ED utilisation increased markedly, particularly among older adults. In-hospital mortality remained low and stable (mean 2.6%). ICU admissions increased from 8.2% to 13.7% of hospitalised patients. Myasthenic crisis accounted for 4.5% of admissions and was strongly associated with ICU utilisation, representing 39% of all ICU stays.

**Conclusions:**

MG care in Spain is undergoing a sustained transition toward ambulatory management without evidence of worsening short-term hospital outcomes. Despite an ageing patient population and rising ICU utilisation, in-hospital mortality remained stable, supporting the safety of outpatient-based MG care models. These findings highlight the need for integrated clinical data to inform future healthcare planning in MG.

**Supplementary Information:**

The online version contains supplementary material available at 10.1186/s42466-026-00485-5.

## Introduction

Myasthenia gravis (MG) is an autoimmune disorder of the neuromuscular junction characterized by fluctuating muscle weakness and variable clinical severity [1, 2]. Over recent decades, several population-based studies have shown a rising prevalence of MG, largely driven by the growing subgroup of late-onset disease and improvements in diagnostic accuracy [3–9]. This epidemiological shift has been accompanied by greater comorbidity burden and increased healthcare utilisation, particularly among older adults. Despite these trends, the organisation of MG care varies considerably across healthcare systems, and contemporary data on patterns of hospital utilisation in Europe remain limited [10, 11].

Hospital admissions for MG provide an important indicator of disease severity, crisis burden and health system performance. Previous analyses from the United States (U.S.) have reported increasing MG-related hospitalisation rates over time, whereas emerging European data suggest more stable or declining trends, reflecting the expansion of specialised outpatient services and the adoption of multidisciplinary care pathways [12]. In addition, the COVID-19 pandemic profoundly altered healthcare activity, with reductions in elective hospitalisations and increased reliance on emergency and ambulatory care, although its specific impact on MG-related healthcare utilisation is not well defined [13–15].

In Spain, MG is managed within a universal healthcare system with extensive provision of specialised outpatient units and day-hospital services. However, no nationwide study has comprehensively examined recent trends in MG-related hospital utilisation across different care modalities, nor evaluated regional variation or the burden of myasthenic crisis at the population level. Understanding these patterns is essential for healthcare planning, resource allocation and the optimisation of neuromuscular care delivery.

The aim of this study was therefore to describe temporal trends, regional patterns and clinical characteristics of MG-related hospital utilisation in Spain between 2016 and 2022, using a nationwide administrative database covering the full adult population. Specifically, we assessed hospital admissions, disease severity indicators (including myasthenic crisis, intensive care unit (ICU) stay and in-hospital mortality), as well as day-hospital activity and emergency department (ED) encounters, to evaluate the population-level safety of evolving MG care models within the Spanish healthcare system.

## Methods

### Data source

For this nationwide, retrospective, population-based study, all hospital episodes with a primary diagnosis of MG were obtained from the Spanish Minimum Basic Data Set (Conjunto Mínimo Básico de Datos, CMBD), maintained by the Ministry of Health of Spain. The CMBD is a mandatory administrative database that records virtually all inpatient admissions and selected outpatient encounters across public and private specialised healthcare facilities, covering a population of approximately 47 million inhabitants. Since 2016, diagnoses and procedures have been coded using ICD-10-ES, the Spanish clinical modification of the International Classification of Diseases, Tenth Revision (ICD-10). ICD-10-ES retains the core diagnostic structure of ICD-10 while incorporating additional national adaptations and extensions to support administrative reporting and procedure coding within the Spanish healthcare system. The registry undergoes yearly updates, although the incorporation of new data occurs with an approximate two-year delay, reflecting the time required for national consolidation and validation processes.

The database includes standardised information on patient demographics, type of healthcare contact, primary and secondary diagnoses, procedures, hospital resource use and discharge outcomes. The primary diagnosis corresponds to the condition established after appropriate evaluation to be chiefly responsible for the healthcare episode. Recording of some outpatient activity types, including hospital day care and ED visits, became mandatory in 2018 [16].

Ethical approval for the study was obtained from the Provincial Research Ethics Committee of Almería.

### Study population

The study population included all hospital episodes with a primary diagnosis of MG recorded between 2016 and 2022 in patients aged ≥ 18 years, identified using ICD-10-ES codes G70.00 (myasthenia gravis without acute exacerbation) and G70.01 (myasthenia gravis with acute exacerbation). The presence of code G70.01 does not necessarily imply myasthenic crisis, and conversely, clinically severe episodes may still be coded as G70.00. Therefore, myasthenic crisis was defined independently using ventilation-related procedure codes, as described below. Eligible episodes were classified as hospital admissions, hospital day care visits, or ED visits.

When an ED visit was immediately followed by a hospital admission, both records were considered part of a single clinical episode, and only the admission was retained for analysis. Only ED visits not resulting in admission were analysed separately. Unique patients were identified through an anonymised linkage based on individual identifiers, date of birth and sex, allowing consolidation of all episodes belonging to the same person throughout the study period.

### Variables

Extracted variables included age, sex, autonomous region, type of contact, type of discharge (including in-hospital death), length of stay, ICU admission and ICU length of stay, secondary diagnoses, and procedures. Hospital admissions were characterized using indicators including number of admissions and unique patients, age distribution, length of stay, ICU admission and ICU stay duration, and in-hospital mortality.

A myasthenic crisis indicator was operationally defined as any MG admission with procedure codes for respiratory ventilation, endotracheal intubation, or tracheostomy [17], independently of the diagnostic code (G70.00 or G70.01). The specific codes used for this definition are provided in Supplementary Table 1 (Online Resource 1).

Comorbidities were identified from secondary diagnostic codes recorded across all episodes for each patient, with duplicates removed. COVID-19 status was assessed using ICD-10-ES code U07.1. Thymus-related conditions and procedures were identified using ICD-10-ES diagnostic and oncologic morphology codes (Supplementary Table 1, Online Resource 1), enabling classification of thymectomy procedures as cancer-related or non-cancer surgeries.

### Statistical analysis

Continuous variables were summarized using mean and standard deviation (SD) or median and interquartile range (IQR), depending on distribution. Categorical variables were expressed as frequencies and percentages. Differences in sex distribution across admission types were assessed using the chi-squared test, while age differences were evaluated using Welch’s ANOVA with Games–Howell. Post hoc comparison tests with Holm correction were conducted for both categories. Logistic regression models examined the association between ICD-10-ES code G70.01, the operational myasthenic crisis definition, and ICU admission.

Annual hospitalization rates per 100,000 inhabitants were calculated overall and by sex, age group (18–44, 45–64, 65–84, ≥ 85 years), and type of admission. Temporal trends were estimated using negative binomial regression models with population offsets. To account for potential structural changes in healthcare recording and utilization patterns after 2018, a breakpoint was incorporated to allow changes in slope from that year onwards. Models included an interaction term between year and post-2018 period to estimate differential trends. Additional models incorporating sex and age group with interaction terms were fitted to obtain stratum-specific annual percent changes. Interaction effects were evaluated using likelihood-ratio tests.

Because recording of hospital day care and emergency department encounters became mandatory only from 2018 onwards, temporal trend analyses for these modalities were restricted to the 2018–2022 period in order to avoid ascertainment bias.

Spatial variation was assessed by estimating period-aggregated hospitalization rates for the 19 Autonomous Communities. Age- and sex-adjusted rates were obtained through indirect standardization using national stratum-specific reference rates. Population counts were retrieved from the Spanish National Institute for Statistics (INE) [18]. Statistical significance was defined as *p* < 0.05. Analyses were performed in R (version 4.5.0).

### Use of AI-assisted tools

ChatGPT (OpenAI, San Francisco, CA, USA) was used solely to improve the clarity and readability of the English text. All content was reviewed and validated by the authors.

## Results

### Overall population description

Between 2016 and 2022, a total of 20,251 hospital episodes with a primary diagnosis of MG were identified from the national registry (ICD-10-ES codes G70.00, *n* = 13,991 [69%], and G70.01, *n* = 6260 [31%]). These corresponded to 6,308 unique patients, of whom 3,953 (62.7%) had a single event during the study period. The mean number of events per patient was 3.6 (range 1–250), with the highest episode counts occurring predominantly among individuals receiving repeated medical day-hospital care. Overall, 49% of patients were female, and the mean (SD) age was 64.3 (17) years.

By type of healthcare contact, 8,066 episodes (40%) were hospital admissions (5,184 unique patients), 10,793 (53%) were hospital day care visits (1,183 unique patients), and 1,392 (7%) were ED visits (696 unique patients).

### Patient characteristics and hospitalization indicators

Among admitted patients (*n* = 5,184), the mean age increased from 61.5 years in 2016 to 65.1 years in 2022, while the proportion of females declined from 54.7% to 46.3%. The overall mean hospital stay was 10.4 days, reaching its highest variability in 2021 (mean 12 days, SD 20.9). ICU admissions rose steadily from 8.2% of hospitalized MG patients in 2016 to 13.7% in 2022, with a mean ICU stay of 11.3 days across the study period.

A total of 362 of 8,066 MG hospital admissions (4.5%) met the criteria for myasthenic crisis. Crisis episodes were markedly more likely to require ICU care than non-crisis admissions (odds ratio 4.8; *p* < 0.0001). Of these 362 crisis episodes, 323 (89.2%) required ICU admission, representing 39% of all ICU stays. Most crisis admissions (317/362, 87.6%) were coded as G70.01, although they represented only 6.6% (317/4,935) of all G70.01-coded admissions. The absolute annual number and proportion of crises increased from 41 (3.8%) in 2016 to 68 (5.9%) in 2022. The global in-hospital lethality was 2.6%, ranging from 2.5% in 2016 to 3.3% in 2022, with a transient peak of 4.2% observed in 2020. All these indicators are summarized in Table 1.

**Table 1:** MG Hospital admissions in Spain, 2016–2022. Patients’ characteristics and Hospitalization indicators by years

Indicator	2016	2017	2018	2019	2020	2021	2022
**Admissions (n)**	1092	1227	1307	1269	1018	998	1155
**Mean age** **(years**,** SD)**	61.5 (17.7)	61.7 (17.5)	62.6 (17.7)	63.2 (17.5)	62.3 (17.4)	63.8 (17.3)	65.1 (16)
**Sex (n**,** %)**							
Female	597 (54.7)	643 (52.4)	718 (54.9)	666 (52.5)	511 (50.2)	532 (53.3)	535 (46.3)
Male	495 (45.3)	584 (47.6)	589 (45.1)	603 (47.5)	507 (49.8)	466 (46.7)	620 (53.7)
**Mean length of stay (days**,** SD)**	9.5 (10.8)	9.6 (13.1)	9.9 (12.4)	10.3 (15)	10.6 (12.7)	12 (20.9)	10.9 (13.3)
**ICU stays (n**,** %)**	89 (8.2)	113 (9.2)	132 (10.1)	129 (10.2)	101 (9.9)	97 (9.7)	158 (13.7)
**Mean ICU length of stay (days**,** SD)**	8.8 (14)	12 (21.5)	8.4 (12.7)	12.9 (21.7)	10.5 (13.2)	14.9 (21.1)	11.4 (14.9)
**Myasthenic crisis (n**,** %)**	41 (3.8)	41 (3.3)	55 (4.2)	57 (4.5)	51 (5)	49 (4.9)	68 (5.9)
**In-hospital deaths (n)**	27	26	32	39	43	34	38
**In-hospital lethality (%)**	2.5	2.1	2.4	3.1	4.2	3.4	3.3

*SD*: standard deviation; *ICU*: intensive care unit

Values are expressed as absolute numbers unless otherwise indicated. Percentages correspond to the proportion of admissions for the given year

### Differences by admission type

**Fig. 1:**
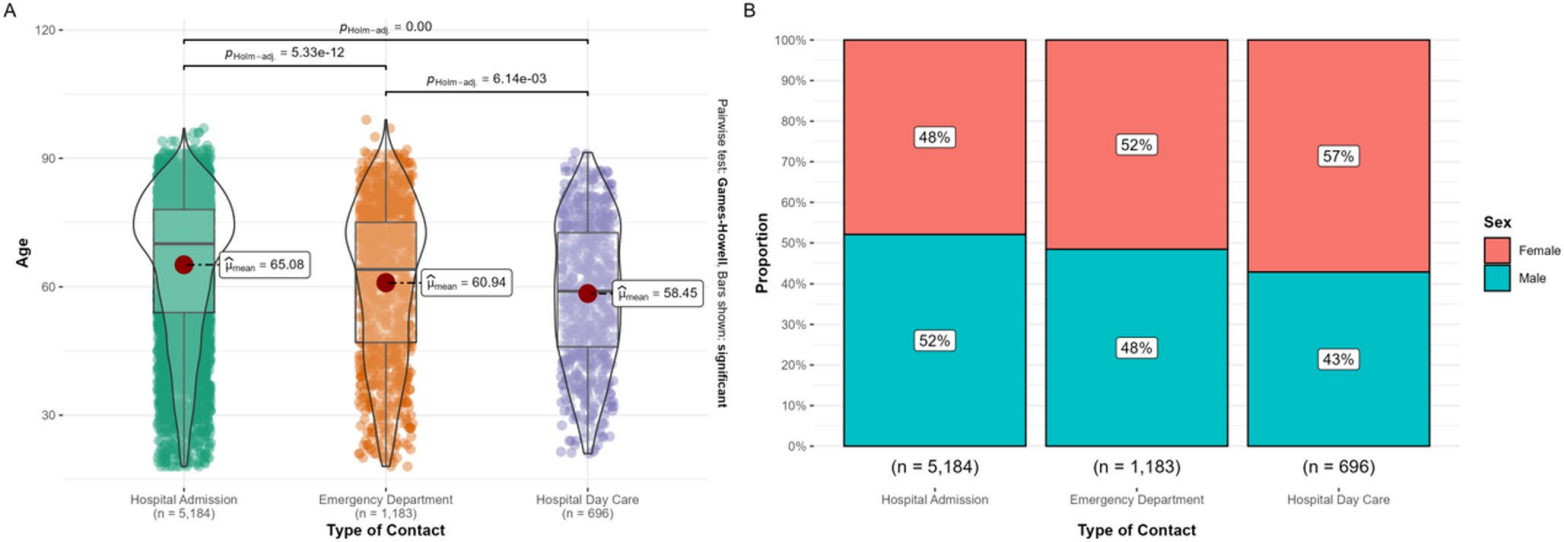
Age and sex distribution across healthcare contact types for myasthenia gravis (2016–2022). **(A)** Violin plots with overlaid boxplots and individual data points showing the age distribution of patients by type of contact (hospital admission, emergency department visit, and hospital day care). Mean ages are displayed for each group. Pairwise comparisons were performed using Welch’s ANOVA with Games–Howell post hoc tests; p-values (Holm-adjusted) for significant contrasts are indicated. **(B)** Stacked bar charts illustrating the proportion of male and female patients across the same contact types, expressed as percentages. Sample sizes for each category are shown below the bars

Significant overall differences in age and sex distribution were observed across admission types (both *p* < 0.001). Compared with hospitalized patients, those treated in hospital day care were younger (mean 58.5 vs. 65.1 years, *p* < 0.001) and more frequently female (57% vs. 48%, *p* < 0.001). ED patients showed intermediate characteristics (mean age 60.9 years; 52% female). Direct comparisons showed that hospital day care patients were younger than patients presenting to the ED (58.5 vs. 60.9 years; *p* < 0.0001), whereas the difference in sex distribution between these two groups did not reach statistical significance (57% vs. 52%; *p* = 0.10). These differences are illustrated in Fig. 1.

### Population rates and temporal trends

Between 2016 and 2022, 8,066 hospital admissions for MG were recorded, corresponding to a mean annual hospitalization rate (HR) of 2.95 per 100,000 inhabitants (range 3.4 in 2018 to 2.54 in 2021). The highest rates were observed in individuals aged 65–84 years (mean 3.58 per 100,000), followed by those aged ≥ 85 years (2.6 per 100,000).

Segmented regression models incorporating a breakpoint in 2018 showed that pre-2018 trends were positive but not statistically significant across strata, with wide confidence intervals reflecting the limited number of pre-2018 observations. In contrast, post-2018 trends demonstrated a significant overall decline in hospital admissions of 5.4% per year (95% CI − 8.4 to − 2.4). Significant post-2018 reductions were observed among females (− 8.8% per year; 95% CI − 13.6 to − 4.0) and in most age groups, including 18–44 years (− 13.8%; 95% CI − 19.7 to − 7.9), 45–64 years (− 5.8%; 95% CI − 10.8 to − 0.8), and ≥ 85 years (− 10.9%; 95% CI − 18.7 to − 3.0).

In contrast, post-2018 trends were not statistically significant among males (− 2.0%; 95% CI − 6.8 to 2.8) or those aged 65–84 years (− 3.0%; 95% CI − 7.5 to 1.5). In-hospital mortality did not show a significant temporal trend in either period. Detailed annual rates and model-derived annual percentage changes are presented in Table 2.

**Table 2:** Annual number of hospital admission episodes for myasthenia gravis between 2016 and 2022, with corresponding rates per 100,000 inhabitants

MG Hospital Admissions	2016	2017	2018	2019	2020	2021	2022	Yearly trend (95% CI)	
	*N* (Rate per 100,000 inhabitants)							Pre-2018	Post-2018
**Total admissions**	1092 (2.85)	1227 (3.21)	1307 (3.4)	1269 (3.28)	1018 (2.6)	998 (2.54)	1155 (2.93)	11.7 (-2.1, 25.4)	-5.4 (-8.4, − 2.4)
**Sex**									
Male	495 (2.67)	584 (3.15)	590 (3.17)	603 (3.21)	507 (2.67)	466 (2.45)	620 (3.24)	16.6 (-5.4, 38.6)	-2 (-6.8, 2.8)
Female	597 (3.03)	643 (3.26)	717 (3.62)	666 (3.34)	511 (2.53)	532 (2.63)	535 (2.64)	7.3 (-14.3, 28.9)	-8.8 (-13.6, -4)
**Age group**									
18–44	215 (0.64)	242 (0.73)	250 (0.77)	222 (0.69)	197 (0.61)	168 (0.53)	136 (0.44)	13.7 (-11.1, 38.5)	-13.8 (-19.7, -7.9)
45–64	338 (1.32)	373 (1.43)	353 (1.33)	360 (1.33)	266 (0.96)	263 (0.93)	338 (1.18)	8.2 (-14.1, 30.4)	-5.8 (-10.8, -0.8)
65–84	470 (3.22)	534 (3.63)	608 (4.08)	585 (3.88)	493 (3.22)	493 (3.18)	606 (3.85)	12 (-8.8, 32.8)	-3 (-7.5, 1.5)
≥ 85	69 (2.55)	78 (2.78)	96 (3.29)	102 (3.38)	62 (1.99)	74 (2.36)	75 (2.33)	8.6 (-27.9, 45)	-10.9 (-18.7, -3)
**Mortality**									
**In-hospital deaths**	27 (0.07)	26 (0.07)	32 (0.08)	39 (0.1)	43 (0.11)	34 (0.09)	38 (0.1)	-3.8 (-57.7, 50)	1.3 (-8.9, 11.4)

MG: Myasthenia gravis; CI: Confidence interval

Annual rates are expressed per 100,000 inhabitants. Temporal trends represent estimated annual percentage changes derived from segmented negative binomial regression models with population offsets, incorporating a breakpoint in 2018 to allow separate slope estimation for the pre-2018 (2016–2017) and post-2018 (2018–2022) periods. Confidence intervals reflect model-based uncertainty

Hospital day care and ED activity can only be assessed from 2018 onwards. Hospital day care activity showed the steepest increase, with rates rising from 2.59 per 100,000 inhabitants in 2018 to 8.6 in 2022, representing a mean annual increase of 27.8% (95% CI 26.8–28.8). This growth was consistent across age and sex groups but more pronounced in females (30.8% vs. 23.9% per year). The 65–84-year group displayed the highest rates and the steepest trend (32.0%, 95% CI 29.5–34.5). ED visits also increased nearly threefold, from 0.37 per 100,000 in 2018 to 1.16 in 2022 (mean annual increase 32.5%, 95% CI 29.7–35.3).

**Fig. 2:**
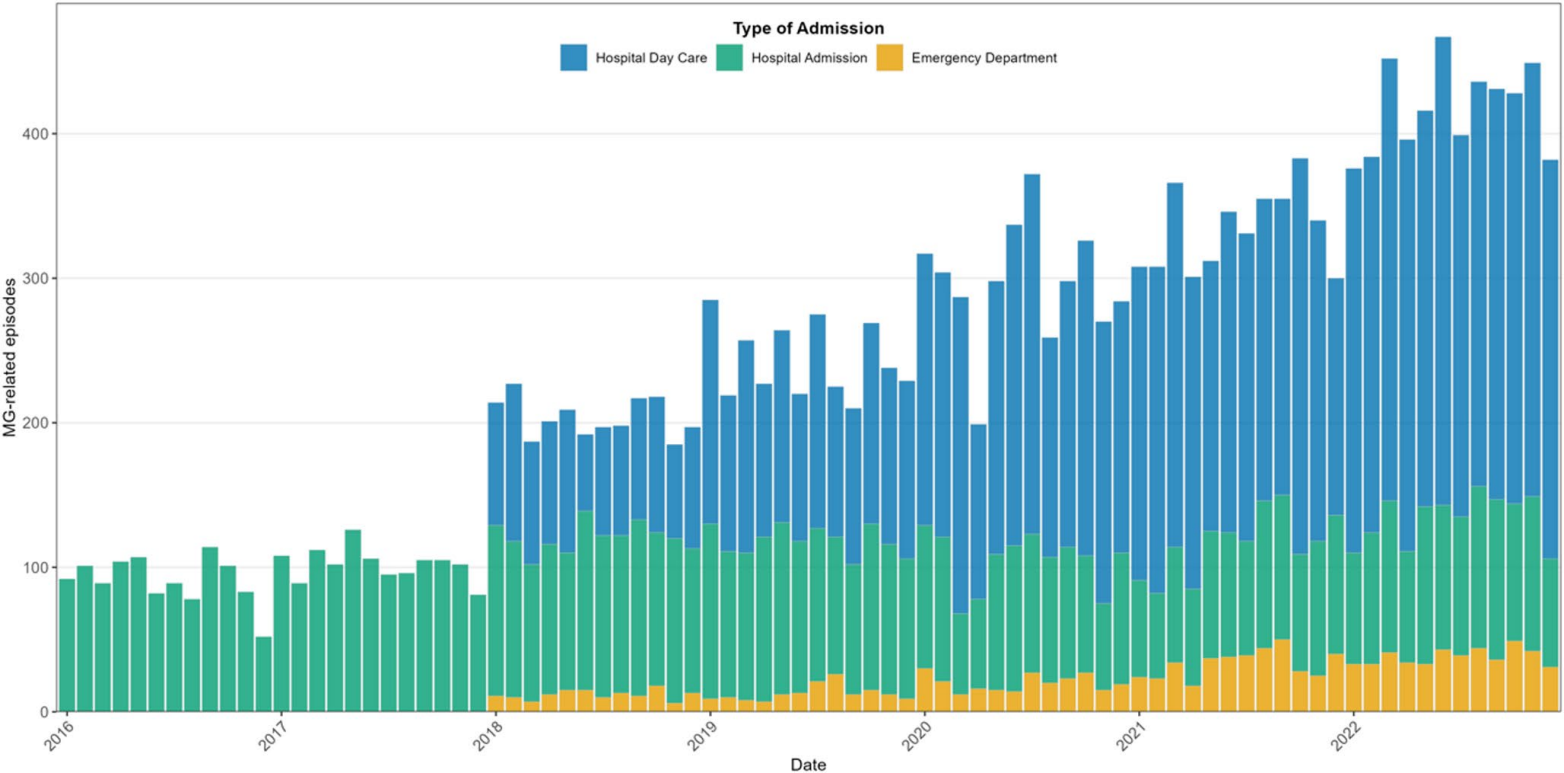
Monthly volume of MG-related hospital utilisation in Spain (2016–2022), stratified by type of healthcare contact. Stacked bar plot displaying the monthly number of MG-related hospital episodes, separated into hospital admissions, medical day-hospital visits and emergency department encounters. The figure illustrates the progressive rise in outpatient activity—particularly day-hospital care—from 2018 onwards, alongside relatively stable or modestly declining inpatient volumes

Data of hospital day care and emergency department are detailed in Supplementary Tables 2 and 3 (Online Resource 1). A combined visual representation of the three care modalities is shown in Fig. 2, highlighting the marked rise in outpatient activity—particularly medical day-hospital care—together with the stabilisation or decline in inpatient admissions.

### Geographical distribution

**Fig. 3:**
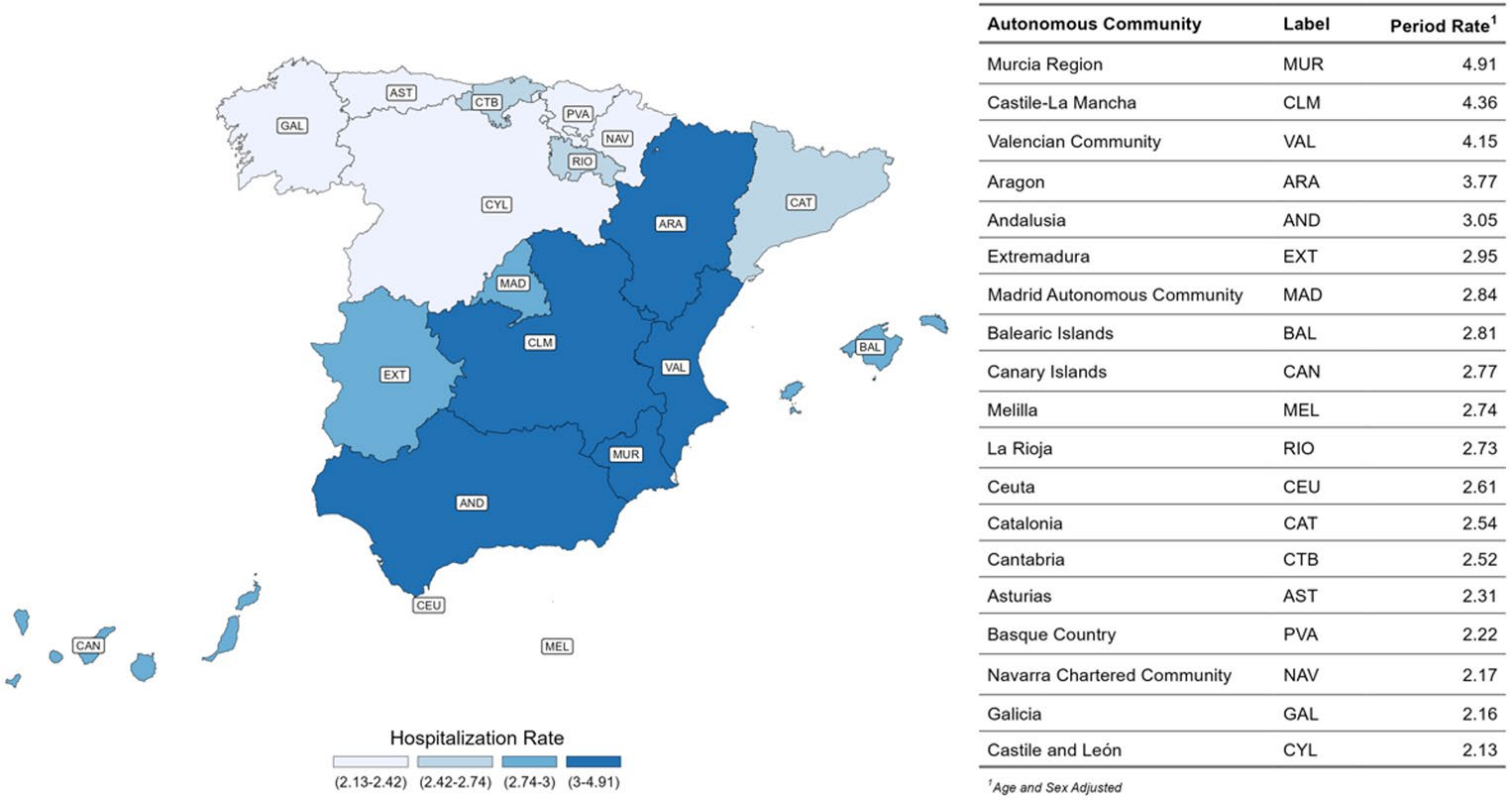
Geographical distribution of myasthenia gravis hospitalization rates across Spanish Autonomous Communities, 2016–2022. Choropleth map displaying period-aggregated, indirectly standardised hospitalization rates per 100,000 inhabitants for each of the 19 Autonomous Communities. Darker shades indicate higher hospitalization rates. The adjacent table summarises the corresponding adjusted rates and regional labels used in the map

Hospitalization rates varied across regions. The highest rates were observed in south-eastern Spain, particularly Murcia (4.91 per 100,000), Castile-La Mancha (4.36), the Valencian Community (4.15), and Aragón (3.77). In contrast, the lowest rates were recorded in Castile and León (2.13), Galicia (2.16), Navarra (2.17), and Asturias (2.31). These data are illustrated in Fig. 3.

### Comorbidities

The most prevalent comorbidities in the study population were essential hypertension (44.4%), hyperlipidaemia (29.1%) and type 2 diabetes mellitus (16%), followed by hypothyroidism, nicotine dependence, obstructive sleep apnoea, and asthma. COVID-19 (U07.1) was recorded as a secondary diagnosis in 97 patients (1.8%). Among admitted patients (*n* = 8,066), 355 had a recorded history of thymic neoplasia—236 malignant thymomas and 119 benign lesions. A total of 312 patients underwent thymectomy. A detailed list of comorbidities, including their absolute frequencies, is provided in Table 3.

**Table 3:** Most frequent comorbidities recorded among hospitalized patients with myasthenia gravis in Spain between 2016 and 2022

ICD-10 Code	Short description	Frequency	Percentage
I10	Essential (primary) hypertension	2,278	44.4
E78.5	Hyperlipidaemia, unspecified	1,495	29.1
E11.9	Type 2 diabetes mellitus without complications	822	16.0
R13.10	Dysphagia, unspecified	510	9.9
H53.2	Diplopia	495	9.6
E03.9	Hypothyroidism, unspecified	422	8.2
F17.210	Nicotine dependence, cigarettes, uncomplicated	407	7.9
G47.33	Obstructive sleep apnoea	400	7.8
E66.9	Obesity, unspecified	389	7.6
N40.0	Enlarged prostate without lower urinary tract symptoms	353	6.9
R47.1	Dysarthria and anarthria	316	6.2
N39.0	Urinary tract infection, site not specified	288	5.6
J96.00	Acute respiratory failure, with hypoxia or hypercapnia	269	5.2
J45.909	Unspecified asthma, uncomplicated	257	5.0
K44.9	Diaphragmatic hernia without obstruction or gangrene	249	4.9

## Discussion

This nationwide, population-based study provides the first comprehensive assessment of temporal trends in hospital utilization and severity-related hospital outcomes for MG in Spain between 2016 and 2022. The findings reveal a progressive reduction in MG-related hospital admissions, driven primarily by declines among women and younger patients, alongside a marked increase in medical day-hospital activity and ED encounters that was consistent across all sexes and age groups. Importantly, this transition toward ambulatory-based care was not associated with worsening short-term outcomes, as reflected by stable in-hospital mortality despite increasing patient age, ICU utilisation and crisis-coded admissions.

While part of the increase in day-hospital and ED activity may reflect the implementation of mandatory reporting in 2018, formal trend analyses for these modalities were restricted to the 2018–2022 period to ensure homogeneous ascertainment. In addition, segmented regression models incorporating a 2018 breakpoint were applied to inpatient admissions to account for potential structural changes in healthcare recording and utilization patterns. Although the limited number of pre-2018 observations resulted in wide confidence intervals, the post-2018 decline in hospital admissions remained statistically significant.

Taken together, the sustained upward trend in ambulatory activity from 2018 onwards—together with the parallel decline in inpatient admissions— is consistent with the broader European [1, 10, 11] transition towards outpatient-oriented management of chronic neuromuscular disorders and supports the hypothesis of an ambulatory-care transition within the Spanish healthcare system.

Our results contrast with recent reports from the U.S., where MG hospitalizations have increased over recent decades. In a large U.S. registry [19] including more than 60,000 admissions, a steady rise in hospitalizations with stable mortality rates (1.5–4.5%) across all age groups was documented, with older adults contributing most substantially to the increase. Similarly, earlier analyses [20] demonstrated rising rates of MG exacerbations leading to hospitalization, attributed to population ageing and increasing comorbidity burden.

In contrast, we observed a significant post-2018 annual decline of 5.4% in hospital admission rates in Spain. This divergence likely reflects structural and organizational differences between healthcare systems. Spain’s universal system has progressively expanded specialised outpatient and day-hospital units capable of administering immunomodulatory therapies, such as intravenous immunoglobulin and plasma exchange, outside the inpatient setting. A comparable stabilization in MG hospitalization rates was recently reported in Germany [10], attributed to improved outpatient care and earlier diagnosis within integrated neuromuscular networks. Together, these findings indicate that although MG prevalence continues to rise globally [21], European healthcare systems are shifting toward ambulatory, multidisciplinary care models that reduce reliance on inpatient admission.

The COVID-19 pandemic introduced an additional inflection point in healthcare utilisation [22]. A sharp decrease in MG hospital admissions during 2020–2021 coincided with increased reliance on day-hospital and ED encounters. This pattern mirrors observations from Turkish, German and Korean cohorts [1, 10, 14], where elective admissions declined and outpatient services expanded as part of pandemic-related reorganization. Although SARS-CoV-2 infection has been described as a trigger for MG exacerbations [22–24], most cohort studies reported non-severe clinical outcomes and low mortality [25–27]. This is consistent with the low proportion of COVID-associated MG admissions observed in our study, and the stable in-hospital mortality during the pandemic years. Together, these data suggest that the pandemic accelerated—but did not initiate—the ongoing shift towards outpatient-based MG management.

Our results reveal progressive ageing among MG patients requiring specialised hospital care, with mean age at admission increasing from 61.5 to 65 years over the study period, and the most pronounced rise in day-hospital and ED utilisation occurring in individuals aged ≥ 65 years. These trends parallel European epidemiological reports describing a growing proportion of older adults within the MG population [3, 28–31]. Older patients are known to exhibit greater respiratory vulnerability and a higher risk of crisis at disease onset [29, 31], although prior studies have suggested that very-late–onset patients may experience comparatively favourable long-term trajectories despite more severe initial presentations. While such clinical distinctions cannot be assessed within our administrative dataset, our findings show that, despite rising ICU use and an increasing number of crisis-coded admissions [32], in-hospital mortality remained low and stable, consistent with other international studies [12, 33, 34]. This stability likely reflects advances in acute management and earlier recognition of clinical deterioration. The relative increase in ICU utilization likely reflects a higher concentration of more severe cases among hospitalized patients rather than a population-level increase in overall disease severity.

The most prevalent conditions among hospitalized MG patients were essential hypertension, hyperlipidaemia, and type 2 diabetes mellitus, followed by hypothyroidism, obstructive sleep apnoea, and asthma. This pattern aligns with prior population-based studies identifying cardiovascular, metabolic, and autoimmune diseases as common comorbidities associated with increased hospital burden and mortality [12, 35]. The high prevalence of vascular risk factors likely reflects both the older age distribution and long-term immunosuppressive treatment effects, particularly chronic corticosteroid use. The coexistence of hypothyroidism and other autoimmune disorders further supports shared immunological predisposition. Age-related immune dysregulation, described in the literature as involving reduced immune responsiveness, chronic low-grade inflammation and increased autoantibody production, may also contribute to the rising proportion of older MG patients and their associated comorbidity burden [36, 37]. The low absolute frequency of thymoma and thymectomy procedures in our cohort is consistent with the predominantly older age profile of the population studied. Although thymoma occurs at a higher relative proportion within the late-onset subgroup compared with early-onset disease, its overall prevalence remains low, which aligns with the epidemiological profile typically associated with late-onset MG [38, 39].

Marked territorial variation in MG hospitalization rates was observed, ranging from 4.9 per 100,000 inhabitants in Murcia to 2.13 in Castile and León, with higher rates clustering in south-eastern Spain. This pattern did not correlate with regional ageing levels (INE data [18]), suggesting that age acts as a predisposing but not determining factor. The observed differences likely reflect variability in healthcare organization, availability of neuromuscular and day-hospital units, and regional differences in CMBD admission and coding practices. Similar interregional variability has been documented in other European studies, emphasizing the influence of organizational factors on MG hospitalization rates [10, 40]. Standardizing admission criteria and expanding outpatient services could help reduce this variability and promote equitable access to specialised care.

These findings have important implications for future service planning. The sustained expansion of day-hospital activity suggests the need to strengthen outpatient neuromuscular units, infusion facilities, and integrated care networks capable of managing increasingly older and multimorbid MG populations within universal healthcare systems. Enhancing coordination across care levels may also help reduce regional variability and ensure equitable access to specialised services.

In addition, future linkage of administrative datasets such as the CMBD with pharmacy dispensing databases and neuromuscular registries could allow validation of diagnostic coding, stratification by treatment intensity, and improved discrimination between active disease and historical MG coding. Such integration would increase confidence that recorded episodes reflect clinically active MG and clarify whether shifts from inpatient to day-hospital care are primarily driven by therapeutic advances or organizational restructuring.

This study has limitations inherent to its retrospective, administrative design. Case identification relied exclusively on primary ICD-10-ES diagnostic codes without clinical validation against medical records, antibody status, electrophysiological findings, or MGFA classification. Therefore, some degree of misclassification (e.g., miscoding of historical MG, ocular versus generalized disease, or coding inaccuracies) is unavoidable. By restricting case identification to primary diagnosis codes, we likely improved specificity for encounters directly attributable to MG, at the expense of potentially underestimating the broader hospital burden associated with MG recorded as a secondary condition.

Myasthenic crisis was defined using ventilation-related procedure codes without clinical confirmation, which may have led to some degree of misclassification. In addition, diagnostic coding of “acute exacerbation” (G70.01) does not necessarily correspond to procedural indicators of crisis, and conversely, clinically severe episodes may still be coded as G70.00. However, such misclassification is likely non-differential over time and therefore unlikely to fully account for the observed temporal trends, although regional coding variability may contribute to geographic differences.

The database lacks detailed clinical information, including antibody status, disease severity scales, and treatment data. Consequently, we could not validate MG diagnoses using disease-specific therapies (e.g., pyridostigmine, corticosteroids, azathioprine, rituximab, eculizumab, IVIG, plasma exchange) nor assess whether evolving hospitalization patterns were associated with specific therapeutic changes. Finally, mandatory reporting of day-hospital and ED activity since 2018 and potential regional differences in admission thresholds may have influenced observed patterns.

## Conclusions

In conclusion, this nationwide study documents a substantial reconfiguration of MG care within the Spanish healthcare system. Following 2018, MG-related hospital admissions showed a significant decline, whereas day-hospital and emergency utilisation increased markedly, consistent with a shift toward ambulatory models of care without compromising short-term outcomes. Despite an ageing patient population and rising rates of ICU use and crisis-coded episodes, in-hospital mortality remained low and stable, underscoring improvements in acute management and crisis recognition. As outpatient-based management continues to expand, future studies integrating clinical, therapeutic and long-term outcome data are required to evaluate the impact of evolving care models and to optimise service planning for an increasingly older and comorbid MG population.

## Supplementary Information

Below is the link to the electronic supplementary material.

Supplementary Material 1.

## Data Availability

The data that support the findings of this study are available from the corresponding author upon reasonable request.
