# Supplementary material for: Trends in hospitalisation, myasthenic crisis and intensive care use in myasthenia gravis: a nationwide population-based study in Spain (2016–2022)
Source: Neurol Res Pract. 2026 Apr 21;8(1):29. doi: 10.1186/s42466-026-00485-5 (PMC13097929; doi:10.1186/s42466-026-00485-5)
Supplement: Supplementary file 1 — Supplementary Material 1. [file 42466_2026_485_MOESM1_ESM.pdf]

Online Resource 1. Supplementary Tables 1–3

Supplementary Table 1. Diagnostic and procedural ICD-10-ES codes applied in the study cohort.

Category	ICD-10-ES Code	Description
**Myasthenia gravis (primary diagnosis)**	G70.00	Myasthenia gravis without crisis
	G70.01	Myasthenia gravis with crisis
**Myasthenic crisis (procedure-based operational definition)**	5A1935Z	Respiratory ventilation < 24 h
	5A1945Z	Respiratory ventilation 24–96 h
	5A1955Z	Respiratory ventilation > 96 h
	0BH17EZ	Endotracheal intubation via natural or artificial opening
	0BH18EZ	Endotracheal intubation via natural or artificial opening, endoscopic
	0B110F4	Tracheostomy, open approach
	0B113F4	Tracheostomy, percutaneous approach
**COVID-19 diagnosis**	U07.1	COVID-19, virus identified
**Thymus-related diagnoses**	C37	Malignant neoplasm of thymus
	D15.0	Benign neoplasm of thymus
	E32.0	Persistent hyperplasia of thymus
	E32.8	Other specified disorders of thymus
**Thymoma-related morphology codes**	858/0	Benign thymoma
	858/0 – 858/6	Malignant forms of thymoma
**Thymectomy procedures**	07TM0ZZ	Total resection of thymus, open approach
	07TM4ZZ	Total resection of thymus, percutaneous endoscopic approach
	07TL0ZZ	Partial excision of thymus, open approach
	07TL4ZZ	Partial excision of thymus, percutaneous endoscopic approach

Supplementary Table 2. Annual number of medical day-hospital episodes for myasthenia gravis between 2018 and 2022, with corresponding rates per 100,000 inhabitants.

**Hospital Day Care**	**2018**	**2019**	**2020**	**2021**	**2022**	**Temporal trend (95% CI)**
	**N (Rate per 100,000 inhabitants)**					
**Total episodes**	994 (2.59)	1495 (3.86)	2294 (5.85)	2607 (6.65)	3403 (8.63)	27.8 (26.8 - 28.8)
**Sex**						
Male	478 (2.57)	618 (3.29)	1002 (5.27)	1165 (6.12)	1294 (6.77)	23.9 (21.8 - 26.1)
Female	516 (2.61)	877 (4.39)	1292 (6.4)	1442 (7.14)	2109 (10.39)	30.8 (28.9 - 32.6)
**Age group**						
18-44	265 (0.81)	437 (1.35)	512 (1.59)	551 (1.74)	658 (2.11)	19.9 (17 - 22.8)
45-64	300 (1.13)	630 (2.33)	1105 (3.99)	1154 (4.1)	1340 (4.68)	27.9 (25.8 - 30.1)
65-84	393 (2.64)	364 (2.41)	595 (3.89)	840 (5.42)	1252 (7.95)	32 (29.5 - 34.5)
≥ 85	36 (1.23)	64 (2.12)	82 (2.63)	62 (1.98)	153 (4.76)	28.2 (20.8 - 35.6)

CI: Confidence interval

Supplementary Table 3. Annual number of emergency department encounters for myasthenia gravis between 2018 and 2022, with corresponding rates per 100,000 inhabitants.

Emergency department	2018	2019	2020	2021	2022	Temporal trend (95% CI)
	N (Rate per 100,000 inhabitants)					
**Total episodes**	141 (0.37)	154 (0.4)	239 (0.61)	400 (1.02)	458 (1.16)	32.5 (29.7 - 35.3)
**Sex**						
Male	59 (0.32)	83 (0.44)	112 (0.59)	203 (1.07)	213 (1.11)	32.9 (27.1 - 38.6)
Female	82 (0.41)	71 (0.36)	127 (0.63)	197 (0.98)	245 (1.21)	32.2 (26.6 - 37.7)
**Age group**						
18-44	29 (0.09)	33 (0.1)	57 (0.18)	94 (0.3)	111 (0.36)	37.8 (29.4 - 46.2)
45-64	44 (0.17)	46 (0.17)	72 (0.26)	113 (0.4)	125 (0.44)	27.8 (20.5 - 35.1)
65-84	62 (0.42)	67 (0.44)	92 (0.6)	168 (1.08)	185 (1.18)	30.1 (24 - 36.3)
≥ 85	6 (0.21)	8 (0.26)	18 (0.58)	25 (0.8)	37 (1.15)	43.6 (27.3 - 59.8)

CI: Confidence interval
